# Papillary thyroid microcarcinoma with contralateral large humerus metastasis and cervical lymph node metastasis: A case report

**DOI:** 10.3389/fonc.2022.924465

**Published:** 2022-08-10

**Authors:** Yi Gong, Shixiong Tang, Wanlin Tan, Liyan Liao, Xiaodu Li, Chengcheng Niu

**Affiliations:** ^1^ Department of Thyroid Surgery, The Second Xiangya Hospital, Central South University, Changsha, China; ^2^ Department of Radiology, The Second Xiangya Hospital, Central South University, Changsha, China; ^3^ Department of Ultrasound Diagnosis, The Second Xiangya Hospital, Central South University, Changsha, China; ^4^ Research Center of Ultrasonography, The Second Xiangya Hospital, Central South University, Changsha, China; ^5^ Department of Pathology, The Second Xiangya Hospital, Central South University, Changsha, China

**Keywords:** papillary thyroid microcarcinoma (PTMC), cervical lymph node metastases, large humerus metastasis, bone metastases, PET/CT, thyroid ultrasonography

## Abstract

**Introduction:**

Papillary thyroid microcarcinoma (PTMC) that metastasizes to bone, especially metastasizes to contralateral humerus with so large mass, is rarely reported before.

**Case report:**

We presented a 50-year-old female patient with a large painful mass in the right humerus for 5 years, presenting with swelling of the right shoulder with limited mobility. Positron emission tomography–computed tomography (PET/CT) showed a large mass in the right humerus, bilateral lung lesions, and enlarged lymph nodes in the right supraclavicular fossa. Right humerus lesion biopsy and immunohistochemical evaluations confirmed that the lesion originated from the thyroid tissue. Then, the thyroid ultrasonography showed a hypo-echoic solid nodule with an irregular taller-than-wide shape in the upper of left thyroid lobe and enlarged lymph nodes with the absence of fatty hilum in the contralateral right IV compartment. The total thyroidectomy and cervical lymph node dissection were undertaken; the histopathology confirmed the diagnosis of PTMC with contralateral cervical lymph node metastasis.

**Conclusion:**

We reported a case of PTMC with contralateral large humerus and cervical lymph node metastasis and demonstrated the PET/CT images of the metastatic large humerus and thyroid ultrasonographic appearances of the PTMC and enlarged cervical lymph node.

## Introduction

Papillary thyroid microcarcinoma (PTMC) is defined as papillary thyroid carcinoma (PTC) measuring equal to or less than 1 cm, which is the common well-differentiated thyroid cancer with an excellent prognosis and extremely low lethality (1). PTMC usually metastasizes to the regional cervical lymph nodes, but metastases to the bones are rarely reported (2, 3). The most common primary sites of metastatic humerus tumors were the breast, myeloma, renal, lung, and prostate carcinomas being the most common sources, and only 2% of these originate from the thyroid (4). To our knowledge, humerus metastasis as an initial presentation of PTMC is almost never reported in the previous literature works, especially as huge as the head of a 3-year-old child. Here, we reported a case of PTMC with contralateral large humerus and cervical lymph node metastasis and demonstrated the positron emission tomography–computed tomography (PET/CT) images of the metastatic large humerus and thyroid ultrasonographic appearances of the PTMC and enlarged cervical lymph node.

## Case report

**Figure 1:**
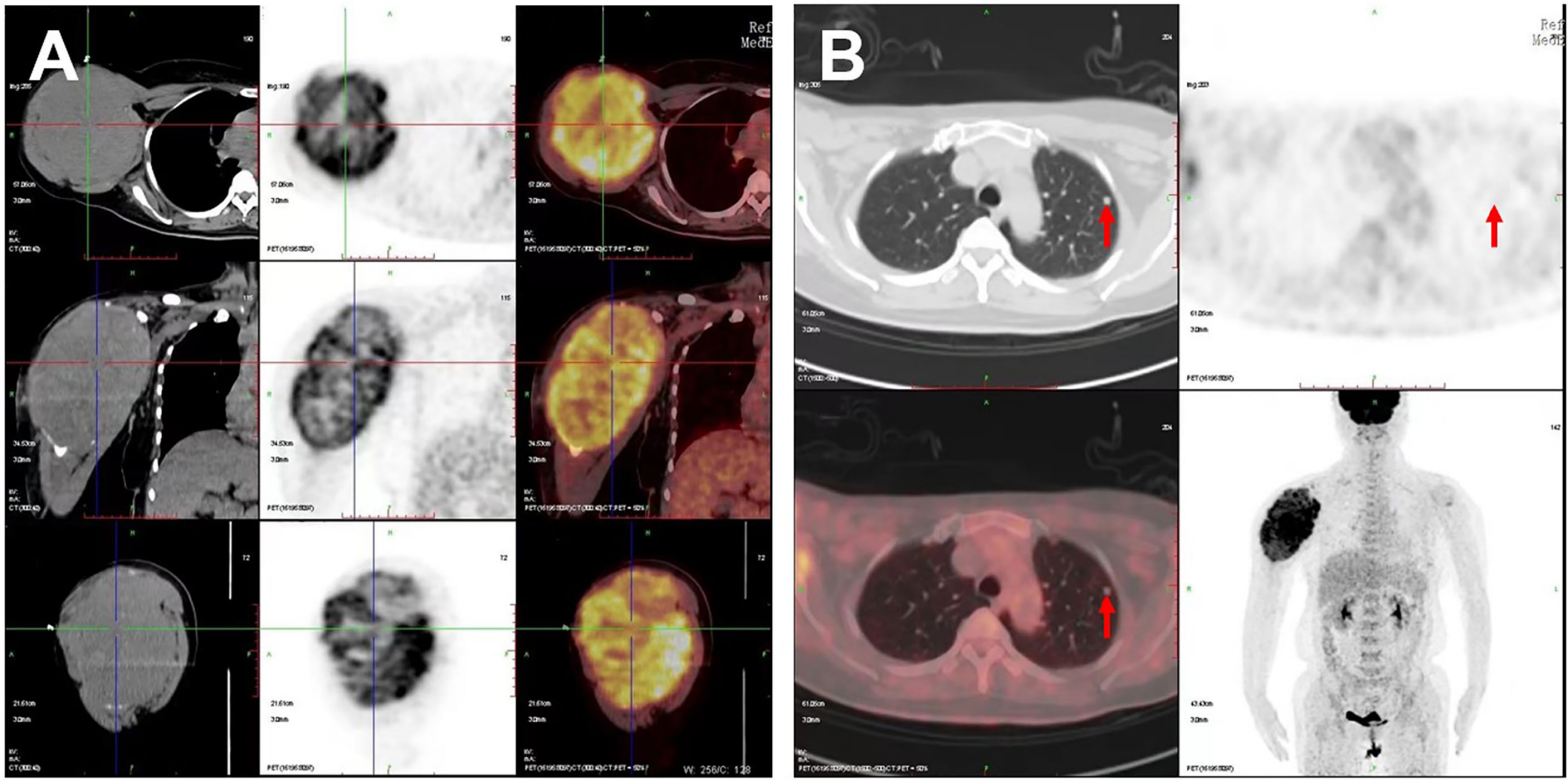
PET/CT images of the patient. Increased 18F-FDG metabolism showed in **(A)** the large right humerus (130 × 115 × 174 mm) on the cross, sagittal, and coronal sections and in **(B)** the left lung (6.5 × 6.0 mm); red arrows indicate the lung lesion.

**Figure 2:**
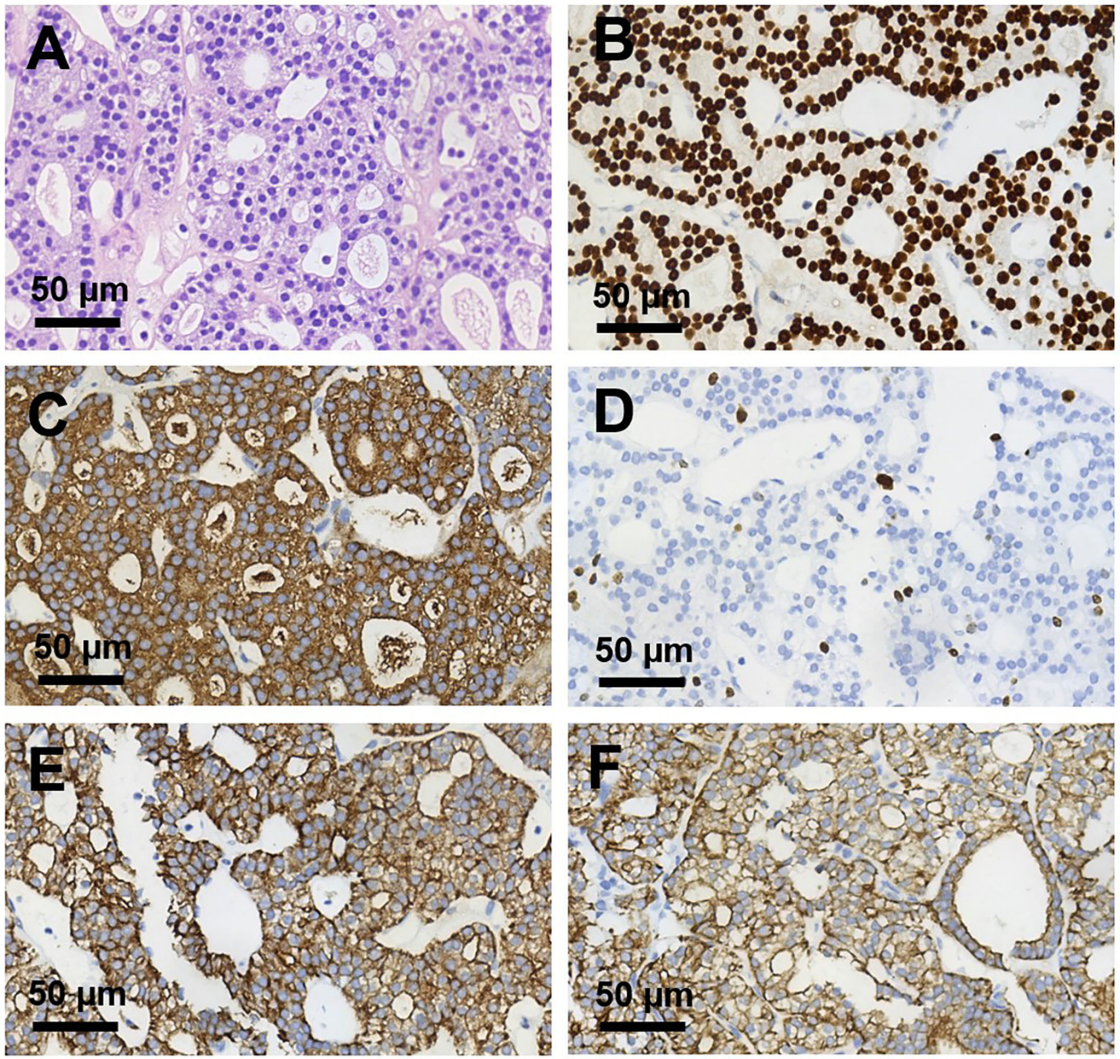
Histopathological sections of right humerus lesion (magnification, ×400). **(A)** H&E staining and **(B–F)** Immunohistochemical staining of **(B)** TTF-1, **(C)** TG, **(D)** Ki 67, **(E)** CK pan, **(F)** CK 7. TTF-1, TG, CK pan, and CK7 were deeply stained (positive); Ki 67 proliferation index was about 10%.

**Figure 3:**
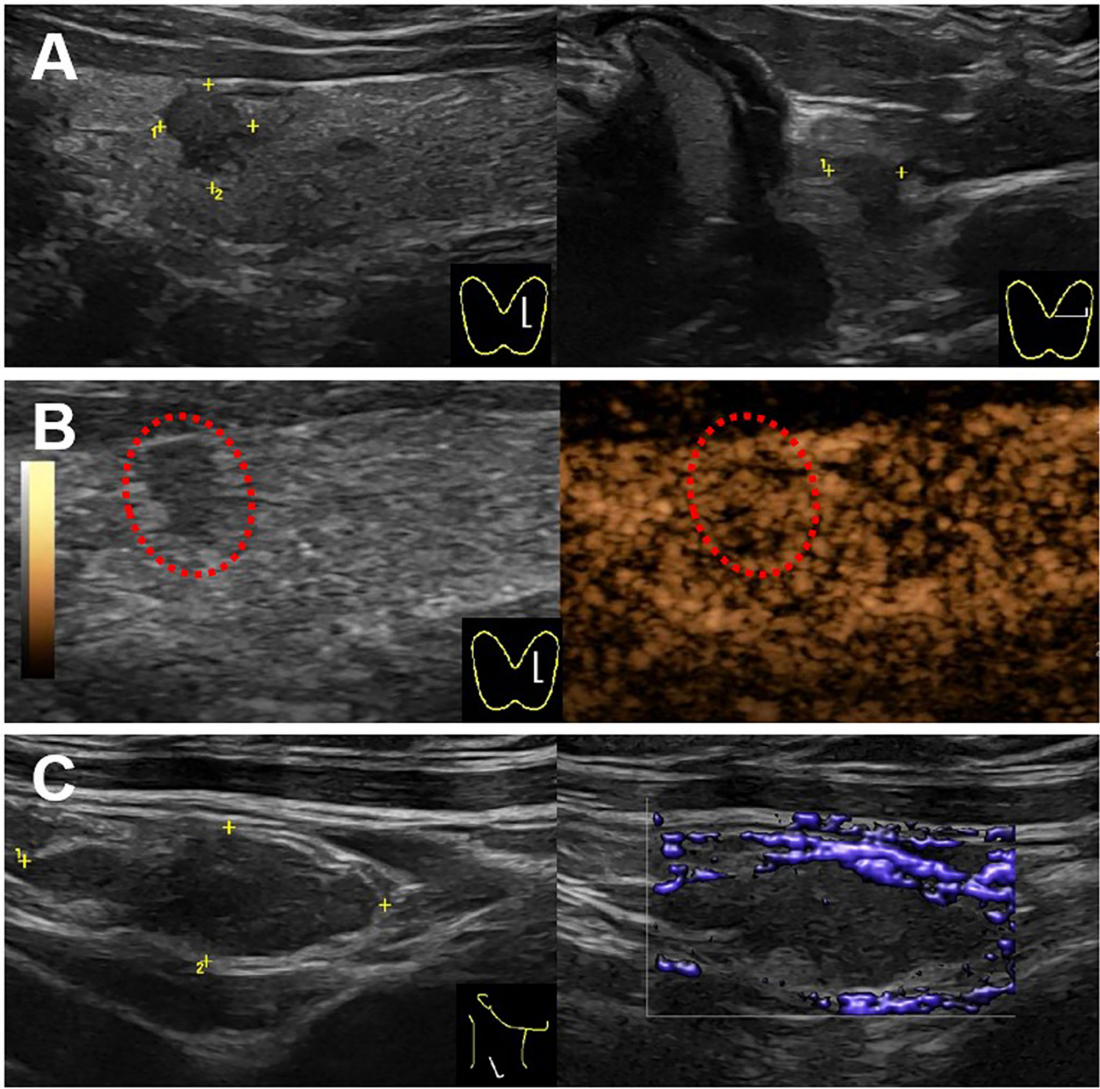
Ultrasound images for the thyroid and cervical lymph node. **(A)** A hypo-echoic solid nodule with an irregular margin and a taller-than-wide shape (4.7 × 3.7 × 5.3 mm) showed in the upper of left thyroid lobe on the gray-mode ultrasonography. **(B)** The nodule showed uneven iso-enhancement on the CEUS mode ultrasonography. **(C)** A swollen lymph node with the absence of fatty hilum (17.4 × 6.6 mm) showed in the right IV compartment of cervical lymph nodes, with no obvious blood flow in the lymph node.

**Figure 4:**
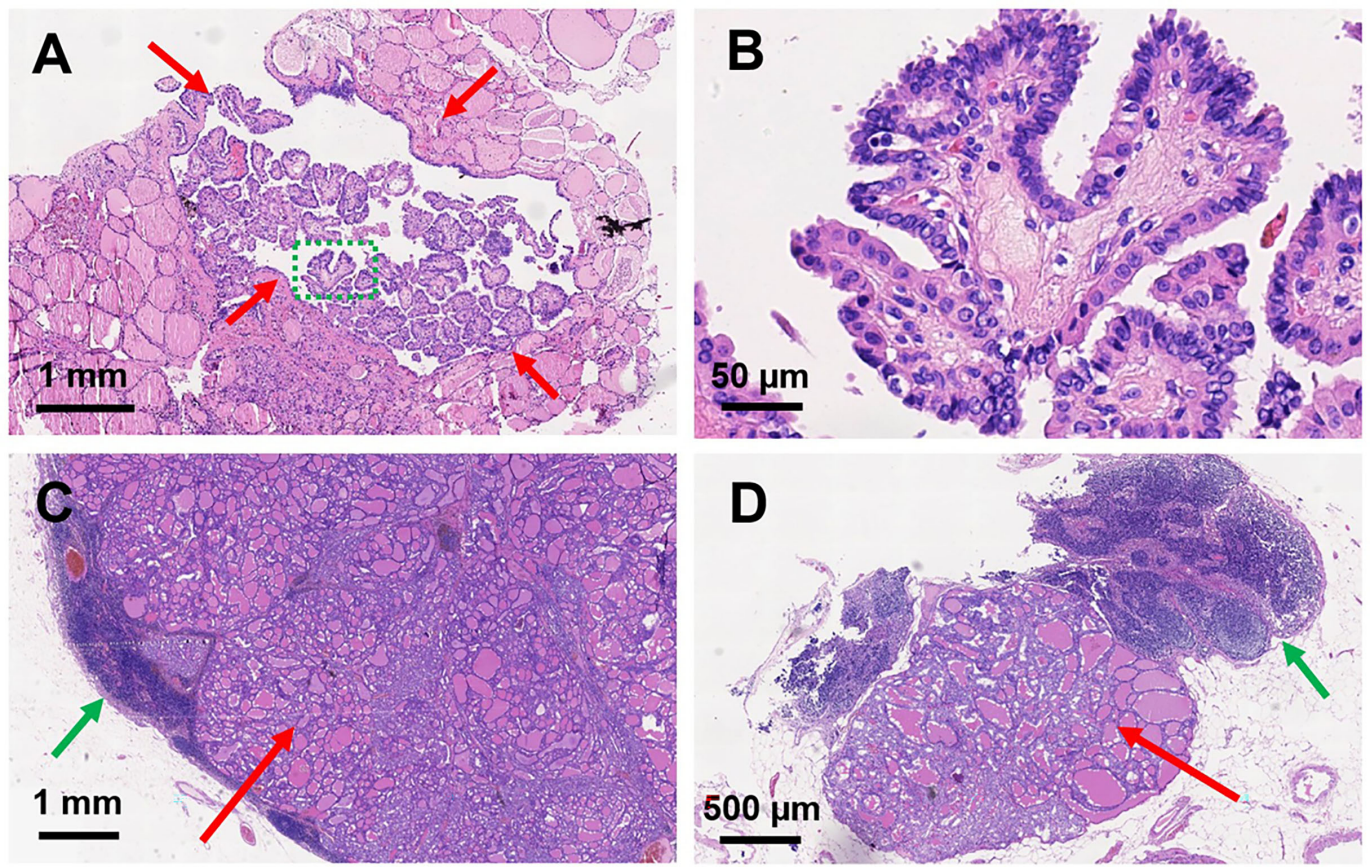
Histopathological sections of papillary thyroid microcarcinoma and metastatic lymph nodes. H&E staining of papillary thyroid microcarcinoma in the left thyroid lobe: **(A)** magnification, × 40; **(B)** magnification, × 400. Red arrows indicate the PTMC; green dashed circle indicates the amplification part in **(B)**. H&E staining of metastatic lymph nodes: **(C)** magnification, ×16; **(D)** magnification, ×34. Red arrows indicate the metastatic thyroid tissue in the lymph nodes; green arrows indicate the normal part of the metastatic lymph nodes.

We presented a 50-year-old female patient with a large painful mass in the right humerus for 5 years, presenting with swelling of the right shoulder with limited mobility. PET/CT with 18F-fluorodeoxyglucose (18F-FDG) showed a large mass in the right humerus (130 × 115 × 174 mm), bilateral lung lesions, and enlarged lymph nodes in the right supraclavicular fossa (**Figure 1**). Right humerus lesion biopsy was carried, and the histopathology of the specimen displayed fistular and sieve distribution with obvious nuclear heterogeneity (**Figure 2A**). The immunohistochemical stains were positive for thyroid transcription factor (TTF-1), thyroglobulin (TG), cytokeratin (CK) pan, and CK7 and negative for hepatocyte (HPC), alpha fetoprotein (AFP), Syn, CgA, special AT-rich sequence-binding protein 2 (SATB2), calcitonin, P53, Napsin A, estrogen receptor (ER), progesterone receptor (PR), and cadual type homeobox gene 2 (CDX2), which indicates that the lesion originated from the thyroid follicular epithelial cells and not from the liver, breast, colon, lung, or thyroid parafollicular cells; Ki67 proliferation index was about 10% (**Figures 2B-F**). Then, the thyroid ultrasonography was carried and revealed a hypo-echoic solid nodule with an irregular taller-than-wide shape (4.7 × 3.7 × 5.3 mm) in the upper of left thyroid lobe; this thyroid nodule has nine points and classified as ACR Thyroid Imaging, Reporting and Data System (TI-RADS) (5). The thyroid nodule showed uneven iso-enhancement on the contrast-enhanced ultrasonography (CEUS), which indicates that the enhancement of thyroid nodule was equal to that of the surrounding tissue. The enlarged lymph nodes with the absence of fatty hilum were displayed in the contralateral right IV compartment of cervical lymph nodes (**Figure 3**). The total thyroidectomy and right lateral cervical lymph node dissection were undertaken; the histopathology confirmed the diagnosis of PTMC with contralateral cervical lymph node metastasis (2/18), indicating two lymph nodes involved in the right IV compartment of cervical lymph nodes, and the total number of the right lateral cervical lymph nodes was 18 (**Figure 4**). Interestingly, the patients had no central cervical lymph node metastasis (0/7), and the BRAF^V600E^ mutation of the PTMC was wild type. According to the eighth edition of the American Joint Committee on Cancer/Tumor Lymph Node Metastasis (TNM) staging system, the patient was in TNM stage IVb (T, N1, of M1) (6).

## Discussion

PTC is the most common malignant carcinoma, with PTMC being one of PTCs with the maximum tumor size of 10 mm, which is considered as the most indolent variant of thyroid cancer (1). The incidence of PTMC is increasing due to the improved diagnostic ultrasonography and fine-needle aspiration biopsy (1). Cervical lymph node metastases are the most metastatic sites for PTMC, whereas the bones and lungs are seldom reported in the literature (7, 8).

Bone metastasis from differentiated thyroid carcinoma is estimated to be 2%–13%; depending on the histologic origin of cancer, follicular thyroid carcinoma (FTC) is more likely to cause bone metastases than PTC (7, 9, 10). The typical metastatic lesions of bone are the spine, ribs, pelvis, and femur; humerus is the most common location of the bone metastases in the upper extremity (11). More than 80% of bone metastases are located in the axial skeleton red marrow, where blood flow is high and tumor cell adhesive molecules are more inclined to bind the tumor cells to migrate (10).

18F-FDG PET/CT has been a predictor of increased aggressiveness and a poor prognosis in many malignant tumors and is helpful in the management of patients with anaplastic and medullary thyroid carcinoma (12). However, it is difficult to identify and estimate the standard uptake value of PTMC on PET/CT due to the tumor size less than 1 cm (13). In this case, increased 18F-FDG metabolism showed on a large mass in the right humerus, bilateral lung lesions, and enlarged lymph nodes in the right supraclavicular fossa, whereas the primary tumor of thyroid without visually identifiable 18F-FDG uptake is missed on PET/CT imaging. For the high 18F-FDG uptake of a mass in the right humerus, the PET/CT images on the cross, sagittal, and coronal sections visually revealed the three-dimensional huge size and high metabolism of the tumor, providing important information for the patient management.

Thyroid ultrasonography is a recommended diagnostic method for thyroid nodes. The typical malignant sonographic features of PTC were solid composition, hypo-echogenicity, irregular margin, presence of calcification, and taller-than-wide shape (5, 14). In this case, this PTMC had four typical malignant sonographic features: solid composition, hypo-echogenicity, irregular margin, and taller-than-wide shape. Hong et al. found that presence of calcification had the predicative for the presence of central compartment lymph node metastases; coincidentally, this case accurately had no central compartment lymph node metastases, but it had contralateral cervical lymph node metastases (15). The mechanism of this jump lymph node metastases is unclear now.

Although PTMC is generally associated with an excellent prognosis and very low mortality rate of 0.5% (16), a study of Orita et al. found that patients with PMTC showed significantly worse survival than patients with standard variant PTC and FTC (9). Another study of Kim et al. found that some PMTC will show aggressive behavior, causing regional or even distant metastases in their earlier presentation, and should not be considered as indolent thyroid carcinoma (17). Weng et al. found that the prognosis of patients with PTMC becomes worse after the development of distant metastases (18). Thus, not all PTMCs are associated with a good prognosis; the mechanism of thyroid metastases in rare sites is unknown, and further research on PTMCs is required, which has a significant impact on patient management.

## Conclusion

In this case, we have reported a case of a large mass in the humerus with swelling of the right shoulder and limited mobility as the first clinical presentation; the humerus lesion biopsy confirmed that it originated from the thyroid tissue. The PET/CT images of the metastatic large humerus and thyroid ultrasonographic appearances of the thyroid nodule and enlarged cervical lymph node were provided. The postoperative histopathology confirmed it as a PTMC with contralateral cervical lymph node metastases. Hence, our case emphasizes that clinically significant metastases can arise from PTMC.

## Data availability statement

The original contributions presented in the study are included in the article/Supplementary Material. Further inquiries can be directed to the corresponding author.

## Ethics statement

This study was reviewed and approved by the Ethics Committee of Second Xiangya Hospital, Central South University, China. The patients/participants provided their written informed consent to participate in this study.

## Author contributions

All authors listed have made a substantial, direct, and intellectual contribution to the work and approved for publication.

## Funding

This project was funded by the National Natural Science Foundation of China (81974267), Science and Technology Innovation Program of Hunan Province (2021RC3033), and Hunan Provincial Natural Science Foundation of China (2022JJ30827 and 2022JJ30806).

## Conflict of interest

The authors declare that the research was conducted in the absence of any commercial or financial relationships that could be construed as a potential conflict of interest.

## Publisher’s note

All claims expressed in this article are solely those of the authors and do not necessarily represent those of their affiliated organizations, or those of the publisher, the editors and the reviewers. Any product that may be evaluated in this article, or claim that may be made by its manufacturer, is not guaranteed or endorsed by the publisher.
